# Development and validation of a white cane use perceived advantages and disadvantages (WCPAD) questionnaire

**DOI:** 10.1186/s40359-023-01282-4

**Published:** 2023-08-30

**Authors:** Zeinab Rasouli Kahaki, Masoud Karimi, Masoud Taherian, Roghayyeh Simi

**Affiliations:** 1 Department of Ergonomics, School of Health, Shiraz University of Medical Sciences, Shiraz, Iran; 2 Research Center for Health Sciences, Institute of Health, Department of Health Promotion, School of Health, Shiraz University of Medical Sciences, Shiraz, Iran; 3 Tehran University, Tehran, Iran; 4 Islamic Azad University of Shiraz, Shiraz, Iran

**Keywords:** Blind, Ergonomics, Visual impairment, White cane

## Abstract

**Background:**

The white cane is globally an important tool in orientation and mobility for blind and visually impaired people, but it is not often used. The goal of this study was to develop and validate the white cane use perceived advantages and disadvantages (WCPAD) questionnaire for detecting effective factors in the use or non-use of canes.

**Method:**

The initial questionnaire items were extracted through semi-structured interviews with 21 blind or severely impaired vision individuals under two main themes, the perceived advantages, and disadvantages of using a white cane. Linguistic validity and writing evaluation with seven experts, face validity with 23 blind persons, content validity ratio (CVR), and content validity index (CVI) were used for assessment of validity. Internal and external reliability assessment was done using Cronbach’s alpha and intra-class correlation coefficient (ICC). Explanatory factor analysis was conducted to identify the factors of the questionnaire; then, corrected item-total correlations, convergent and discriminant validity, and Confirmatory Factor Analyses (CFA) were done, and model fit indices were reported. A total of 320 blind and severe visual impaired individuals (138 males and 182 females) participated in the study. The final questionnaire contained 12 questions in perceived advantages and 21 in perceived disadvantages.

**Results:**

Three factors (social participation, mobility, and culture) extracted for perceived advantages which explained 67.95% of the total variance, Cronbach-α for the three extracted factors was between 0.75 and 0.91. Four factors (social, safety, ergonomics, and family) for perceived disadvantages were extracted which explained 50.98% of the total variance. Cronbach-α for the four extracted factors was from 0.75 to 0.88. CFA confirmed the final models for perceived advantages and disadvantages.

**Conclusion:**

WCPAD questionnaire has good reliability and validity, and the factors obtained from factor analysis can measure the reasons for using or otherwise of white canes.

## Introduction

Vision serves as a primary source of stimulus and plays a crucial role in how individuals interact with the world. When compared to other senses, it plays an essential role in gaining control of an individual on the ability to move and discover the environment, thereby facilitating direct communication with the surrounding environment and allowing the individual gain valuable experiences which facilitate adaptation to the environment [1–3].

In 2020, there were 43.3 million blind persons in the world, and 295 million people had severe to moderate visual impairments. 90% of these cases were reported in developing countries, and Iran is the 16^th^ country in this ranking [4].

The blind and visually impaired people are encountered with many challenges in their lives, including education, employment, and social interactions [5, 6]. In all these areas, they have difficulty finding their way. Wayfinding, which is defined as navigating independently, safely, and easily from one intended place to another, is perhaps the main challenge in the life of blinds [7, 8]. For blind and partially sighted people, alternative methods are used to compensate for the lack of vision and help them move [9]. One of the most common and appropriate mobility assistive devices is the white cane [10, 11]. The use of white canes is considered the oldest, simplest, and most widespread method of helping visually impaired people to be independent and move [12]. Not only does the white cane help people with visual impairments recognize the obstacles, ups, and downs, but it also implies that others are aware of their presence [13, 14].

Although the white cane is globally an important tool in orientation and mobility for blind and visually impaired people to move, they are reluctant to use it due to their negative attitude about the white cane [15].

Users’ acceptance of assistive technology is an important factor that contributes to the use and satisfaction of devices, and reduction of the risks of their abandonment. Santos et al. [13] in a qualitative study on visually impaired European students concluded that devices with no negative symbolism which have modern aesthetics designs such as smart glasses were better accepted by the participants than traditional white canes [13]. According to Hersh’s study [15], different factors such as stigma, white cane use adaptation and acceptance, safety concerns, cane use as a symbol of blindness, and formal and informal training may influence the visually impaired people’s intention to use white cane. Another study conducted by Maidenbaum et al. [16] revealed that although there were several reasons why many visual impaired individuals avoid using a white cane, the main factors were fear of striking people or objects with it, hitting obstacles at a height that the white cane did not recognize, and social stigma.

Despite the limited number of studies conducted on the attitudes of blind people toward white cane, it is noted that the idea of being labeled, feeling of shame and embarrassment caused by using a cane, as well as the views of the community about visually impaired people are the most important causes of the negative attitude about the use of white cane [13, 15].

Since positive or negative attitudes are powerful predictors of behaviors, it is essential to examine the attitudes of visually impaired people towards the use of white cane; it can reveal if they behave appropriately while moving and finding their way or not and helps to take appropriate measures to change their attitudes and encourage them to use the white cane.

To help the researchers and policymakers obtain accurate information about the attitudes of visually impaired people about the use of white cane, they need to use a valid and reliable tool. Since few studies have been conducted in this field and the authors could not find a valid tool to use, using a cane in the individual and social lives of the blind is important, and a comprehensive and complete investigation of the reason why the cane is used or is not is needed, the present study aimed to develop a valid and reliable tool, called white cane use perceived advantages and disadvantages (WCPAD) questionnaire.

## Method

This methodological study was conducted in Iran (2021). It was approved by the Ethics Committee of Shiraz University of Medical Sciences, Shiraz, Iran (Code of Ethics: IR.SUMS.REC.1399.1336) and conducted in accordance with the declaration of Helsinki [17]. The study consisted of two phases: the development phase and validation phase.

### Development phase

In this phase, to develop a questionnaire items pool, we conducted 10–30 min semi-structured face-to-face or phone interviews with 21 (10 males and 11 females) blind or severely impaired vision individuals. The interviews were done from April to May 2021; the participants were selected using the purposive sampling method disregarding whether they used or did not use a white cane. At first, the participants were asked about their demographic characteristics such as age, job status, education level, and using or not using a white cane. Then, they were asked to fully describe their motivations and reasons for using or not using a cane. Questions were also asked about the role of family and community in shaping their attitudes towards white cane. The interview guide is represented in Table 1. All the interviews were digitally recorded and transcribed verbatim. The data were coded and analyzed through directed content analysis. Through this method, content analysis was done in an organized way, based on the predetermined constructs [18]. According to the directed content analysis, the responses were coded, categorized, and placed under two main themes: the perceived advantages and perceived disadvantages of using a white cane. At the end of this phase of the study, 35 and 19 items for perceived disadvantages and perceived advantages of using white cane were extracted, respectively.

**Table 1:** Interview guide used for interviews in development phase

1. How old are you?
2. How long have you had a vision impairment?
3. Do you use a white cane?
• If yes, how long have you been using a cane? and why?
• If not, have you ever used a white cane? why don’t you use a cane now?
4. What are the advantages of using a white cane for you?
5. Based on your experiences, what are the disadvantages of using a white cane?
6. Which factors make you to use or not use a white cane?
7. How much does your family play a role in using or not using white cane? Please explain
8. How do people in the community treat you when you have a white cane in your hand?

### Validation phase

The following steps were taken to assess the psychometric properties of the questionnaire.

#### Step 1: linguistic validity and writing evaluation

In this step, a panel of seven experts consisting of two health promotion professionals; two psychologists, one of whom was blind; and three ergonomists reviewed and confirmed the questionnaire.

#### Step 2: face validity

The face validity of the questionnaire was confirmed qualitatively, using 23 available samples of blind people. They were asked to assess the items in terms of problems, ambiguity, idioms, and grammar, and the requested corrections were made based on the opinions of the expert panel described in the previous step.

#### Step 3: content validity

For assessment of the content validity, content validity ratio (CVR) and content validity index (CVI) were calculated based on Lawshe et al. [18] and Waltz and Bussel’s [19] guidelines, respectively. For this purpose, the prepared questionnaire was assessed by 15 health promotion professionals, ergonomists, psychiatrists, and 3 blind people. The items which had a Content Validity Ratio (CVR) above 0.5 and a content validity index (CVI) above 0.79 remained in the questionnaire, and the rest of them were deleted. Finally, 26 items remained in the disadvantages (Table 2) and 18 items in the advantages sections (Table 3).

**Table 2:** Item statistics for reliability analysis: CVR, CVI, and ICC for disadvantages of white cane use

Item	CVR	CVI	External reliability			
			Test Mean (SD)	Retest Mean (SD)	ICC	*P*
1. I do not use a cane because of the inappropriate attitude of people	0.88	1	1.96 (1.24)	2.00 (1.22)	0.99	0.001
2. I do not want others to notice my blindness	0.77	1	1.90 (1.13)	1.76 (1.17)	0.95	0.001
3. If I hold a cane, others may feel sorry for me	0.77	0.94	2.57 (1.32)	2.66 (1.42)	0.90	0.001
4. Using a cane hurts my pride	0.77	0.88	2.04 (1.20)	2.09 (1.22)	0.95	0.001
5. Using a cane destroys my dignity and lowers my social status	0.77	0.83	1.80 (1.07)	1.90 (1.09)	0.96	0.001
6. I'm worried that people will abuse me when I hold a cane	0.66	0.88	1.80 (0.75)	1.85 (1.01)	0.79	0.001
7. I'm worried that others will make fun of me when I hold a cane	0.77	0.88	1.85 (1.06)	1.71 (1.00)	0.94	0.001
8. I have not received enough training on moving and wayfinding	1	1	2.81 (1.47)	2.80 (1.40)	0.95	0.001
9. Most people are not familiar enough with the condition of the person holding a white cane	0.77	0.94	3.80 (1.24)	3.90 (1.22)	0.97	0.001
10. My family does not like me holding a cane	1	1	1.85 (1.10)	1.81 (1.07)	0.75	0.001
11. My family prefers me to go out with someone rather than holding a cane	0.88	1	2.57 (1.39)	2.61 (1.53)	0.80	0.001
12. I can easily find my way without a cane	0.88	0.94	2.09 (0.94)	1.71 (0.96)	0.86	0.001
13. I cannot trust the cane in moving and wayfinding	0.55	0.88	2.14 (1.01)	2.28 (1.05)	0.89	0.001
14. Using a cane has little effect on reducing the risk of an accident	0.77	0.94	2.09 (1.18)	2.14 (1.15)	0.95	0.001
15. The cane is an extra and cumbersome tool	0.88	0.88	1.86 (0.91)	2.04(1.24)	0.85	0.001
16. Using a cane does not give me a sense of security	0.66	0.88	2.09 (1.04)	2.19 (1.25)	0.82	0.001
17. I would rather go out with someone than walk alone with a cane	0.88	1	2.42 (1.16)	2.33 (1.23)	0.94	0.001
18. The obstacles and the uneven sidewalks and streets make the use of a cane ineffective for me	0.66	0.88	3.00 (1.26)	2.95 (1.43)	0.88	0.001
19. It is not possible to use a cane in crowded places	0.88	1	2.81 (1.12)	2.86 (1.19)	0.95	0.001
20. Using a cane does not help me much in crossing the street	1	0.94	2.95 (1.39)	2.81 (1.40)	0.86	0.001
21. Using a cane does not affect reducing the number of accidents on the street	0.66	0.94	2.47 (1.21)	2.52 (1.29)	0.98	0.001
22. A cane can only mark obstacles on the ground	0.66	0.88	4.00 (1.22)	3.77 (1.33)	0.88	0.001
23. The canes we use weigh a lot	0.66	0.88	2.57 (1.07)	2.57 (1.02)	0.91	0.001
24. The canes we use are not designed properly	0.77	0.88	2.62 (0.92)	2.90 (1.13)	0.76	0.001
25. The available canes are not foldable or easy to carry	0.55	0.83	2.19 (0.87)	2.47 (1.12)	0.75	0.001
26. I have pain in my wrist when I use a cane	0.55	0.88	2.28 (1.05)	2.24 (1.13)	0.94	0.001

**Table 3:** Item statistics for reliability analysis: CVR, CVI, and ICC for advantages of white cane use

Item	CVR	CVI	External reliability			
			Test Mean (SD)	Retest Mean (SD)	ICC	*P*
1. The white cane is a symbol of blindness/low vision and helps others understand how to treat us	0.88	1	4.38 (0.59)	4.47 (0.60)	0.75	0.001
2. I consider the cane as an aid, like glasses	0.77	0.94	4.33 (0.79)	4.47 (0.81)	0.82	0.001
3. Using a cane improves the culture of proper treatment of the blind in society	0.88	1	3.90 (1.18)	3.85 (1.19)	0.88	
4. Using a cane, I can appear more often in society	0.88	1	4.14 (0.91)	4.28 (0.78)	0.75	0.001
5. Using a cane helps me find my way more easily	0.88	1	4.14 (0.91)	4.33 (0.96)	0.90	0.001
6. The white cane helps increase my independence in doing my daily activities	0.88	1	4.38 (0.80)	4.62 (0.74)	0.76	0.001
7. I feel more secure with the help of a cane	1	1	4.04 (1.24)	4.19 (1.16)	0.89	0.001
8. Using a cane prevents me from falling	0.77	0.94	4.04 (1.02)	4.09 (1.17)	0.90	0.001
9. The cane helps me identify the existing obstacles such as stairs, potholes, and ditches	0.88	1	4.57 (0.50)	4.52 (0.60)	0.44	0.021
10. Using a cane makes others push/jostle me less	0.66	0.88	4.04 (1.02)	4.14 (0.91)	0.92	0.001
11. Using a cane makes me less likely to bump against people	0.77	0.88	4.29 (0.96)	4.09 (1.04)	0.92	0.001
12. The cane helps me cross the street	1	1	3.66 (1.23)	3.57 (1.20)	0.97	0.001
13. Using a cane helps me maintain a better body posture and alignment	0.55	0.83	3.95 (0.92)	3.61 (1.11)	0.75	0.001
14. When I appear in society with a cane, people admire and respect me	0.55	0.94	3.14 (1.19)	3.09 (0.94)	0.76	0.001
15. I’m not stressed when I walk with a cane and as a result my self-confidence increases	0.88	1	3.90 (0.94)	3.80 (0.98)	0.95	0.001
16. In the event of an accident, using the cane, I will have the protection of the law	0.88	0.94	3.66 (1.11)	3.62 (1.28)	0.85	0.001
17. The cane increases my courage to move independently	0.77	0.94	4.33 (0.79)	4.28 (1.10)	0.77	0.001
18. I have been used to having a cane since I was a child	0.66	0.88	2.14 (1.35)	2.19 (1.32)	0.99	0.001

#### Step 4: reliability assessment

External reliability of the questionnaire was established by calculating the intra-class correlation coefficient (ICC) using 2-way mixed-effects model in a test–retest reliability analysis on a pilot sample of 30 participants with two weak intervals. Based on the recommendations of Bujang and Baharum [20], considering the α < 0.05, minimum power (β) of at least 90%, with the expected ICC value of at least 0.5, a minimum sample size of 30 was considered sufficient for this step. Values less than 0.5, between 0.5 and 0.75, between 0.75 and 0.9, and greater than 0.90 were considered as poor, moderate, good, and excellent reliability, respectively [21]. The results are presented in Tables 2 and 3 for the perceived disadvantages and advantages of using a white cane, respectively.

#### Step 5: participant recruitment and data collection

Based on the suggestions of Tinsley and Kass [22] who consider a maximum of 300 participants as a suitable sample size for factor analysis, a total of 320 blind and visually impaired people were enrolled in the study. The subjects were selected through the snowball sampling method throughout the country. Participation in the survey was completely voluntary. Inclusion criteria included more than 10- year-old males or females who lived in Iran, were blind or visually impaired severely, and needed to use a white cane. Participants who did not respond to all items of questionnaires were excluded from the study.

Data collection tools consisted of a demographic information form (age, sex, education level, marital status, using white canes or not), and two questionnaires that assessed the perceived disadvantages (26 Items) and perceived advantages (18 items) of using a white cane. All the items were measured by a five-point Likert scale (strongly agree to strongly disagree). The instrument was prepared in a Google docs form and administered to the participants through e-mail or instant messaging apps such as WhatsApp. As to the participants who could not respond to the questionnaire by mobile phone, the researcher called them, and the questionnaire was completed by phone interview. The survey was conducted from July to September 2021.

#### Step 6: construct validity

In this step, explanatory factor analysis using an elaboration likelihood model with Promax rotation was conducted to identify the factors of the questionnaire; then, corrected item-total correlations and confirmatory factor analyses (CFA) were used, and model fit indices were reported. The internal consistency of the questionnaire was determined using Cronbach’s alpha coefficient. All the analyses were conducted using SPSS 24 and IBM AMOS 24 software. Ultimately, convergent and discriminant validity of the two perceived advantages and disadvantage scales were assessed using correlation matrix between the total scores of the two scales and their subscales.

## Results

Overall, 182 females (mean age 30.48 ± 10.15) and 138 males (mean age 31.12 ± 11.36) participated in the study. The mean age of the total participants was 30.76 ± 10.02 (range: 10–61 years). Table 4 displays the frequency distribution of the respondent’s demographic characteristics.

**Table 4:** Frequency distribution of the respondents’ demographic characteristics

Variable	N	%
Marriage		
Married	88	27.5
Single	232	72.5
Education level		
Ninth grade and less	55	17.2
Diploma	72	22.5
Bachelor degree	107	33.4
Master’s degree	76	23.1
PhD	12	3.8
Using white cane		
Yes	154	48.1
No	166	51.9
The onset of visual impairment		
Since birth	187	58.4
Gradual	93	29.1
Suddenly	40	12.5

The normal distribution of all the items of the white cane use perceived disadvantage questionnaire was assessed and confirmed by skewness (-1.53, 1.03) and kurtosis (-1.35, 2.78) indices. Elaboration likelihood model analysis with Promax rotation was conducted on the 26 items of the perceived disadvantages of using a white cane. Five items with low factor loadings were removed, giving a 21-item solution that explained 50.98% of the total variance. Item-total correlation coefficients were from 0.52 to 0.73, and Cronbach-α for the four extracted factors was from 0.75 to 0.88 (Table 5).

**Table 5:** Factor analysis of 21-item white cane disadvantages questionnaire

*Item N*	Factor 1 Social	Factor 2 Safety	Factor3 Ergonomic	Factor4 Family	Item total correlation
1	0.76				0.717
2	0.74				0.684
3	0.77				0.639
4	0.77				0.729
5	0.67				0.642
6	0.65				0.603
7	0.78				0.729
13		0.53			0.631
14		0.82			0.682
16		0.76			0.692
17		0.43			0.547
18		0.42			0.577
19		0.51			0.639
20		0.77			0.611
21			0.76		0.571
23			0.73		0.599
24			0.75		0.559
25			0.61		0.535
26			0.54		0.522
10				0.68	0.714
11				1.07	0.714
Eigenvalue	3.242	4.609	1.901	1.463	
%Of Variance	14.736	20.948	8.642	6.652	
Cronbach α	0.88	0.86	0.75	0.76	

The normal distribution of all the items of the white cane use perceived advantages questionnaire was assessed and confirmed by skewness (-1.53, 0.70) and kurtosis (-0.55, 2.41) indices. Elaboration likelihood model analysis with Promax rotation was conducted on the18 items of the perceived disadvantages of using a white cane. Six items with low factor loadings were removed, giving a 12-item solution that explained 67.95% of the total variance. Item-total correlation coefficients were from 0.34 to 0.78, and Cronbach-α for the three extracted factors was between 0.75 and 0.91 (Table 6).

**Table 6:** Factor analysis of 12 item white cane advantages questionnaire

Item N	Factor1 Social participation	Factor2 Mobility	Factor3 Culture	Item total correlation
4	0.77			0.755
5	0.92			0.766
6	0.89			0.776
7	0.84			0.775
15	0.58			0.693
17	0.70			0.763
10		0.82		0.630
11		0.74		0.641
12		0.70		0.468
1			0.73	0.516
3			0.60	0.476
16			0.70	0.338
Eigenvalue	6.03	1.24	0.88	
% Of Variance	50.26	10.33	7.35	
Cronbach α	0.91	0.75	0.63	

**Fig. 1:**
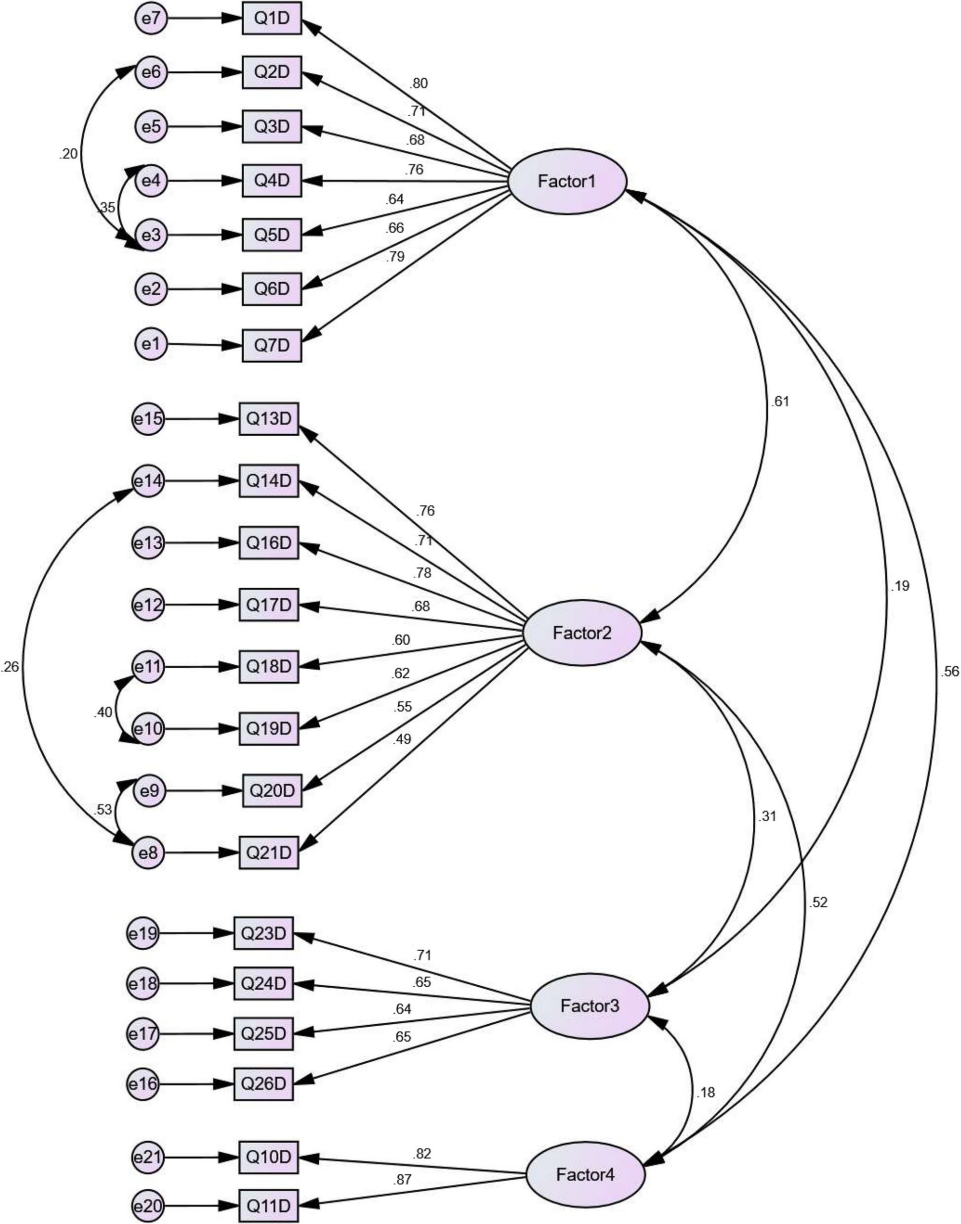
Confirmatory factor analysis of four factors of white cane disadvantages of WCAD questionnaire (*N* = 320)

**Fig. 2:**
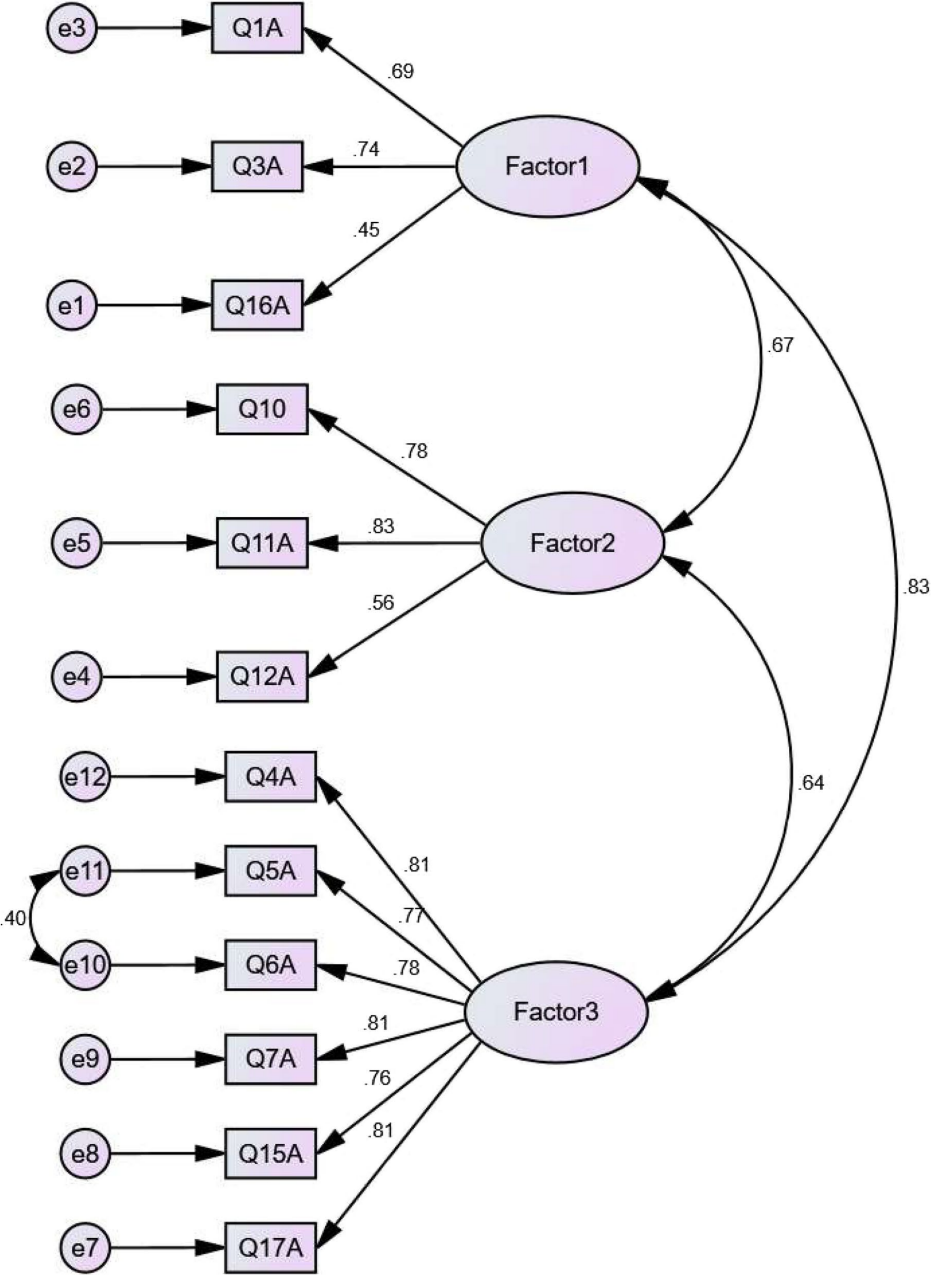
Confirmatory factor analysis of three factors of white cane advantage of WCAD questionnaire (*N* = 320)

Confirmatory Factor Analysis was conducted to evaluate the Construct validity of the four factors of white cane use perceived disadvantages (Fig. 1) and three factors of white cane use perceived advantage (Fig. 2) questionnaires.

The chi-square/degrees of freedom ratio (${}^{{x}^{2}}\!\left/ \!{}_{df}\right.$), Normed fit index (NFI), the comparative fit index (CFI), Tucker-Lewis’s index (TLI), adjusted goodness-of-fit index (AGFI), Parsimonious Normed Fit Index (PNFI), and the root means the square error of approximation (RMSEA) were used to confirm the Goodness-of-fit of the model (Table 7).

**Table 7:** Result of confirmatory factor analyses

Model	x⁄df	NFI	CFI	TLI	AGFI	PNFI	RMSEA
Disadvantage	2.32	0.87	0.92	0.91	0.86	0.74	0.06
Advantage	2.77	0.93	0.96	0.94	0.89	0.71	0.07
Acceptable ranges	< 3	> 0.9	> 0.9	> 0.9	> 0.80	> 0.50	< 0.08

As shown in Table 8, there was moderate to strong positive correlations between the total scores of perceived disadvantages of white cane use and its subscales (*r* > 0.6), and strong positive correlation between perceived advantages of white cane use and its subscales (*r* > 0.7); on the other hand, a moderate negative correlation was seen between the total scores of perceived disadvantages and advantages of white cane use (*r* = -0.552). Also, there were negative correlations between the subscale of perceived disadvantages and advantages of white cane use. These findings confirm the convergent and discriminant validity of the two scales.

**Table 8:** Pearson’s correlation coefficients between total scores of perceived disadvantages and advantages of white cane use and their subscales

	Perceived Disadvantages					Perceived Advantages			
	Total	Social	Safety	Ergonomic	Family	Total	Social P	Mobility	Culture
Perceived Disadvantages									
Total	1								
Social	0.835^a^	1							
Safety	0.856^a^	0.543^a^	1						
Ergonomic	0.563^a^	0.213^a^	0.433^a^	1					
Family	0.628^a^	0.478^a^	0.435^a^	0.195^a^	1				
Perceived Advantages									
Total	-0.552^a^	-0.398^a^	-0.578^a^	-0.279^a^	-0.291^a^	1			
Social P	-0.565^a^	-0.446^a^	-0.594^a^	-0.213^a^	-0.310^a^	0.931^a^	1		
Mobility	-0.395^a^	-0.207^a^	-0.465^a^	-0.305^a^	-0.159^a^	0.786^a^	0.573^a^	1	
Culture	-0.375^a^	-0.276^a^	-0.359^a^	-0.224^a^	-0.230^a^	0.799^a^	0.635^a^	0.528^a^	1
Mean	57.60	17.77	19.81	14.38	5.63	46.21	23.29	10.89	12.03
SD	15.40	7.30	6.42	3.82	2.64	8.78	5.14	2.74	2.31

^a^correlation is significant at the 0.01 level (2-tailed)

## Discussion

The present study was conducted to develop and validate a questionnaire for assessing the attitudes of people with visual impairments about white cane use perceived advantages and disadvantages (WCPAD). To the best of the authors’ knowledge, a questionnaire in this regard has not been designed so far, and this is the first study that has developed a tool to measure the reasons for using or not using a white cane.

The content validity ratio (CVR) for the remaining items in both perceived advantages and disadvantages constructs of the questionnaire was more than 0.55; according to Lawshe’s criteria and the number of experts panel (18 experts), this indicates appropriate content validity [18]. The content validity index (CVI) for the remaining items in the questionnaire on both perceived advantages and disadvantages sections was higher than 0.83, which was appropriate based on Waltz and Basel’s criteria [19].

The internal consistency of the questionnaire was checked using Cronbach’s alpha coefficient which was obtained in the two parts of the perceived advantages and disadvantages of the final questionnaire (0.90 and 0.91, respectively), indicating good internal reliability [23].

The external reliability of the questionnaire was checked by the test–retest method on 30 blind people who were eligible to participate in the study with an interval of one week, and the intra-class correlation coefficient (ICC) was calculated [24]. ICC is one of the most suitable reliability coefficients. Based on the 95% confidence interval of the ICC estimate, values less than 0.5, between 0.5 and 0.75, between 0.75 and 0.9, and greater than 0.90 indicated poor, moderate, good, and excellent reliability, respectively [21]. The range of ICC for all the remaining items in both questionnaires of the advantages and disadvantages of using a white cane was 0.75 to 0.99, showing good and excellent external reliability of the questionnaire.

The results of exploratory factor analysis showed that the 21 items of the questionnaire on the disadvantages of using a white cane were placed in four social (7 items), safety (7 items), ergonomics (5 items), and family factors (2 items). Confirmatory factor analysis showed the appropriate goodness of fit indices of the model [25, 26].

Based on the results of exploratory factor analysis, 12 items of the questionnaire on the advantages of using white cane were placed on three factors including social participation (6 items), mobility (3 items), and culture (3 items). Confirmatory factor analysis showed the appropriate goodness of fit indices of the model [25, 26].

In a qualitative study carried out in 2015, Hersh analyzed the factors encouraging and prohibiting the use of canes in a six-component model including stigma, safety concerns, acceptance and adaptability in using canes, cane as a symbol of blindness, and provision of formal and non-formal education [15]. Considering blind people as weak individuals and the feeling of pity from society, non-acceptance of disability conditions, experience of a sense of inferiority and embarrassment, people’s fear of getting hurt, weak mobility and orientation skills, and white cane forms are other factors that have been mentioned in a few studies [13, 15, 27]. In the current study, many of the factors and items obtained are in line with those mentioned in other studies [28, 29].

### Limitations

This is the first study that developed and validated a tool to examine the advantages and disadvantages of using a white cane, and the help of the blind community throughout the country was considered in this process. However, according to the cultural conditions of Iran, its generalizability to other countries and cultures may be limited, and it should be used with caution; it is suggested that its psychometric features should be evaluated in other cultures. In addition, since both EFA and CFA were conducted by the same participants, due to the special conditions of the participants and limited access to them, our findings and extracted models may be at risk of overfitting. So, the model may perform poorly when applied to new data or the broader population.

## Conclusion

The results of this study showed that WCAD questionnaire had good reliability and validity, and the factors obtained from factor analysis could be used to determine the reasons for using and not using the canes well; it can be used in future studies as a reliable and efficient tool to investigate the attitudes of the blind community about the use of white cane. It should be noted that the findings of the current research are the first tools in this field and more extensive research should be done in continuation of this work.

## Data Availability

The data can be made available upon reasonable request from the Corresponding author.
